# Ultrasound-Based Tomographic Imaging Reconstruction and Synthesis Methods: a Scoping Review

**DOI:** 10.1007/s10278-025-01674-5

**Published:** 2025-10-14

**Authors:** Daniel de Wilde, Aron Alakmeh, Olivier Zanier, Raffaele Da Mutten, Anatol Aicher, Gustav Burström, Erik Edström, Adrian Elmi-Terander, Stefanos Voglis, Luca Regli, Carlo Serra, Victor E. Staartjes

**Affiliations:** 1 Machine Intelligence in Clinical Neuroscience & Microsurgical Neuroanatomy (MICN) Laboratory, Department of Neurosurgery, Clinical Neuroscience Center, University Hospital Zurich, University of Zurich, Zurich, Switzerland; 2 Department of Neurosurgery, Cantonal Hospital of Lucerne, Lucerne, Switzerland; 3 Department of Clinical Neuroscience, Karolinska Institutet, Stockholm, Sweden; 4 Capio Spine Center Stockholm , Löwenströmska Hospital, Upplands-Väsby, Sweden; 5 Department of Neurosurgery, Region Örebro County, Örebro University Hospital, Örebro, Sweden

**Keywords:** Artificial intelligence, Deep learning, Image translation, Synthetic tomography, Machine learning, Ultrasound

## Abstract

Ultrasound (US) imaging is valued for its safety, affordability, and accessibility, but its low spatial resolution and operator dependence limit its diagnostic capabilities. Tomographic imaging modalities like computed tomography (CT) and magnetic resonance imaging (MRI) offer high-resolution 3D visualization but are cost-prohibitive and complex. Ultrasound-based tomographic imaging aims to combine the advantages of both modalities, potentially democratizing access to advanced imaging. A scoping review was conducted following PRISMA-SR guidelines. Articles were identified through searches in PubMed MEDLINE, Embase, Scopus, and arXiv from inception to July 2025. Eligibility criteria included full-text original studies focused on ultrasound-based tomographic imaging generation or reconstruction methods. Out of 8256 identified articles, 86 met the inclusion criteria. Studies examined four imaging modalities: photoacoustic tomography (36%), ultrasound computed tomography (36%), 3D reconstruction (20%), and synthetic imaging (7%). Deep learning algorithms (67%) were the most common, followed by iterative reconstruction algorithms (9%), and other methods. The breast (17%), brain (16%), and blood vessels (14%) were the most studied anatomical regions. This review highlights advancements in ultrasound-based tomographic imaging, driven by deep learning innovations. Despite progress, the field is still in its infancy, and challenges remain in clinical adoption, particularly in standardization and validating performance. Future research should focus on improving algorithm efficiency, generalizability, and validation.

## Introduction

Ultrasound (US) imaging is a widely used clinical tool that employs high-frequency sound waves to produce real-time, two-dimensional (2D) images of internal body structures. It allows dynamic assessment of anatomy, organ function, and interventional procedures such as catheter placement [1]. The mechanism is based on sound waves being emitted via electrical stimulation of a piezoelectric crystal within the probe, reflecting as echoes when interacting with tissues. These echoes are captured by the transducer, converted into electrical signals, and reconstructed into 2D images [1, 2]. US is favored in many clinical scenarios due to its non-invasive nature, absence of ionizing radiation, affordability, and accessibility. However, US has substantial limitations: its spatial resolution is lower than other imaging modalities, the field of view is restricted, and image acquisition relies heavily on operator expertise [3]. These factors can compromise diagnostic and therapeutic accuracy, particularly for complex or deep-seated anatomical structures [3].

Tomographic imaging modalities such as computed tomography (CT) and magnetic resonance imaging (MRI) provide high-resolution, three-dimensional (3D) visualization [4–7]. CT offers rapid and detailed imaging, ideal for trauma, oncology, and surgical planning, but exposes patients to ionizing radiation [4, 5]. In contrast, magnetic resonance imaging (MRI) avoids radiation exposure and excels at soft tissue contrast but is time-consuming, costly, and unsuitable for patients with certain metallic implants or pacemakers [6, 7]. Furthermore, the substantial cost, logistical requirements, and operational complexity of tomographic imaging systems limit their availability in low-resource settings, such as primary care, obstetrics, critical care units, and low- and middle-income countries (LMIC) [8].

To address these gaps, the concept of leveraging ultrasound data to generate tomographic-like imaging has gained increasing attention [9–13]. This approach combines the safety, affordability, and accessibility of ultrasound with the high-resolution, 3D advantages of tomographic imaging, potentially democratizing access to advanced imaging [9, 10]. Early efforts in this domain began in 1977, when ultrasound computed tomography (USCT) was introduced for breast imaging [14]. Since then, progress has been limited.

A range of image reconstruction methods has been developed to bridge this gap, each with distinct assumptions and applications. Traditional analytic methods, such as delay-and-sum (DAS) and filtered back-projection algorithms, are computationally efficient but suffer from poor image resolution and limited ability to handle complex wave propagation [15]. Building on these approaches, physics-informed reconstruction methods embed prior knowledge, such as acoustic speed distributions, into the reconstruction pipeline [16].

However, recent advancements in artificial intelligence, particularly generative deep learning methods such as generative adversarial networks (GANs), convolutional neural networks (CNNs), and diffusion models, have substantially enhanced the capability of image synthesis. These innovations now enable the reconstruction and generation of new imaging modalities such as synthetic tomographic imaging, USCT, photoacoustic tomography (PAT), and 3D reconstruction [9, 17–19].

Despite these advancements, there remains a lack of comprehensive literature reviewing the methodologies and technologies used for generating tomographic imaging from ultrasound data. This scoping review systematically assesses the current state of the art, highlights key methods, identifies knowledge gaps, and outlines future research directions. By synthesizing these insights, we aim to accelerate the development and clinical adoption of ultrasound-based tomographic imaging.

## Methods

### Overview

Due to the limited existing literature available, a scoping review was deemed more appropriate than a systematic review. This review adhered to PRISMA-SR guidelines and followed the methodological framework proposed by Arksey and O’Malley throughout the process [20].

### Search Strategy

PubMed/MEDLINE, Embase, Scopus, and arXiv were searched from inception up to June 29, 2024, to identify eligible papers. A follow-up literature search was performed to identify studies added to the databases between June 2024 and July 2025. The search strategy was as follows:

(“ultrasound” OR “ultrasonography” OR “ultrasonic imaging” OR “sonography” OR “sonographic” OR “sonogram”) AND (“MRI” OR “magnetic resonance imaging” OR “CT” OR “computed tomography” OR “CAT scan” OR “tomography” OR “tomographic imaging”) AND (“deep learning” OR “CNN” OR “convolutional neural network” OR “GAN” OR “generative” OR “generation” OR “synthesis” OR “synthetic”).

References were extracted and uploaded into a web-based systematic review tool (Rayyan.ai) to facilitate duplicate removal and independent screening [21]. Title and abstract screening, full-text review, and final article selection were carried out independently by two reviewers (DDW and AA). All disagreements were resolved upon discussion with a third reviewer (VES). Reference lists of the included articles were also screened, resulting in further study inclusions.

### Selection Criteria

Inclusion criteria were as follows:

1. Studies focusing on the synthetic generation of tomographic imaging from 2D or 3D ultrasound data;
2. Studies describing reconstruction algorithms for ultrasound-based tomographic imaging.

Only full-text, original articles in English were considered. Exclusion criteria comprised: (1) reviews or non-technical publications, such as commentaries or editorials; (2) studies on ultrasound-tomographic imaging registration algorithms; (3) studies generating ultrasound images from tomographic imaging; (4) studies combining ultrasound with existing tomographic data for generation or reconstruction of tomographic imaging; (5) articles not published in English.

### Data Extraction

A predefined data extraction sheet was used to systematically chart relevant information from the included studies. Extracted data included (a) study location (country), (b) year of publication, (c) primary objective, (d) type of ultrasound imaging, (e) type of tomographic imaging, (f) reconstruction method, (g) sample size, (h) dataset details, and (i) anatomical region.

### Data Synthesis and Reporting

A descriptive analysis was performed due to the heterogeneity of the included studies, with no assessment of performance metrics. The primary objectives were to identify key methodologies for generating or reconstructing tomographic images from 2D or 3D ultrasound data, extract critical insights, analyze historical and current trends, and outline future directions for ultrasound-based tomographic imaging research. Graphs were created using GraphPad Prism (version 8.0.2, GraphPad Software Inc., Massachusetts, United States), RStudio (version 1.4.1106, Posit, PBC, Massachusetts, United States) with the gganatogram package [22], and Excel (vrsion 2408, Microsoft, WA, USA).

## Results

### Overview

**Fig. 1:**
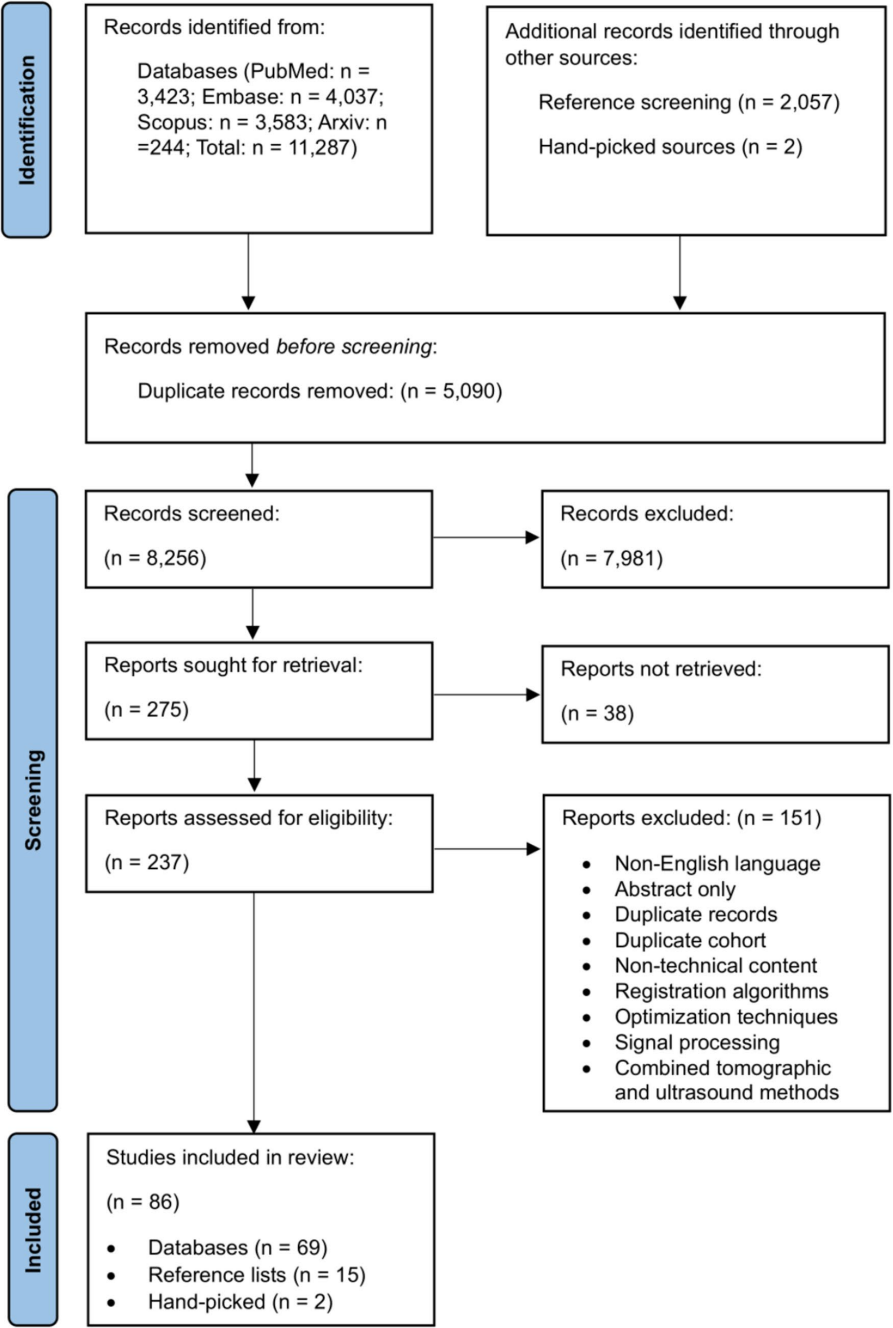
PRISMA Flowchart illustrating study selection process

A total of 8256 unique articles were sourced through database searches and reference lists. Following title and abstract screening, 275 articles were selected for full-text review. Of these, 38 were excluded due to unavailable full texts, four were exact duplicate publications, two were duplicate cohorts, one was non-English, and 144 did not meet the inclusion criteria. Excluded studies primarily focused on registration algorithms, hybrid imaging systems, signal processing, or were non-technical. Ultimately, 86 articles were included, comprising 69 articles sourced through database searches, 15 from reference lists (Fig. 1), and two from manual selection.

### Bibliographical Aspects

#### Publication Years

**Fig. 2:**
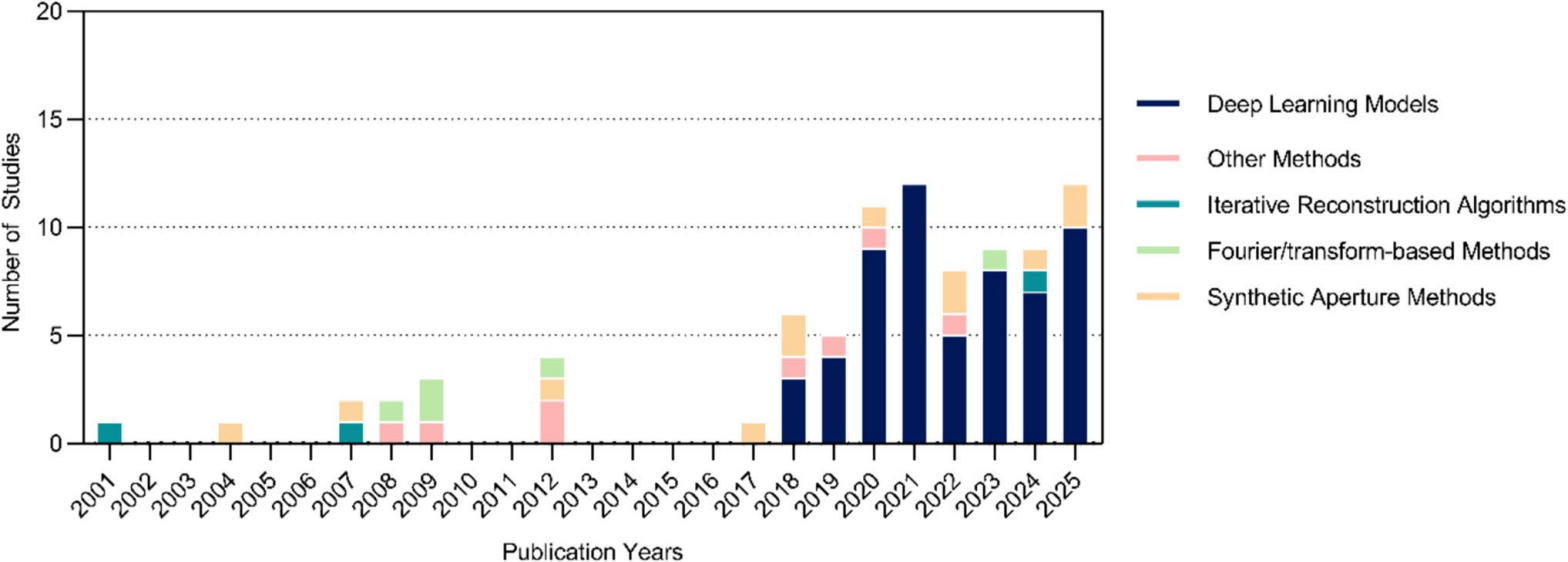
Distribution of included studies by year of publication and categorized according to the reconstruction method used

Only four studies (*n* = 4, 5%) were published prior to 2008. Between 2008 and 2017, 12% (*n* = 10) of studies were published. The majority of studies (*n* = 60, 70%) were published between 2018 and 2024, with a notable peak in 2021 (*n* = 12, 14%). As of July 2025, 12 studies (14%) had been published in 2025 (Fig. 2).

#### Geographical Distribution

**Fig. 3:**
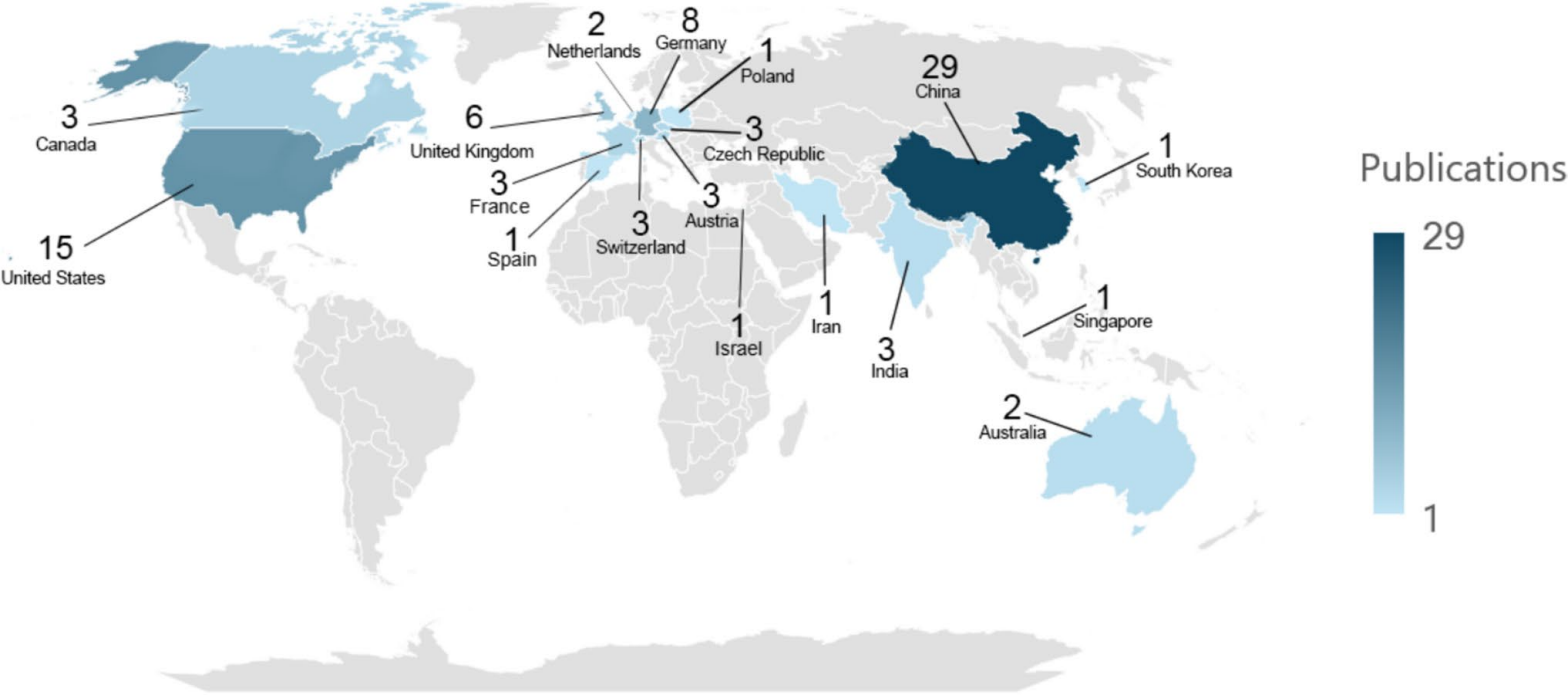
Global distribution of author affiliations

Of the included studies, 36 (42%) were conducted at Asian institutions, 30 (35%) at European institutions, 18 (21%) at North American institutions, and two (2%) at Australian institutions (Fig. 3).

#### Publication Venue

Most studies (*n* = 65, 76%) appeared in peer-reviewed journals, while 21 (25%) were preprints. The majority of studies were published in biomedical engineering journals (*n* = 41, 47%), followed by computer science journals (*n* = 13, 15%), engineering journals (*n* = 12, 14%), clinical journals (*n* = 6, 7%), physics-focused medical journals (*n* = 6, 7%), interdisciplinary medical-computer science journals (*n* = 5, 6%), and pure physics journals (*n* = 3, 3%).

### Methodological Aspects

#### Anatomical Region

**Fig. 4:**
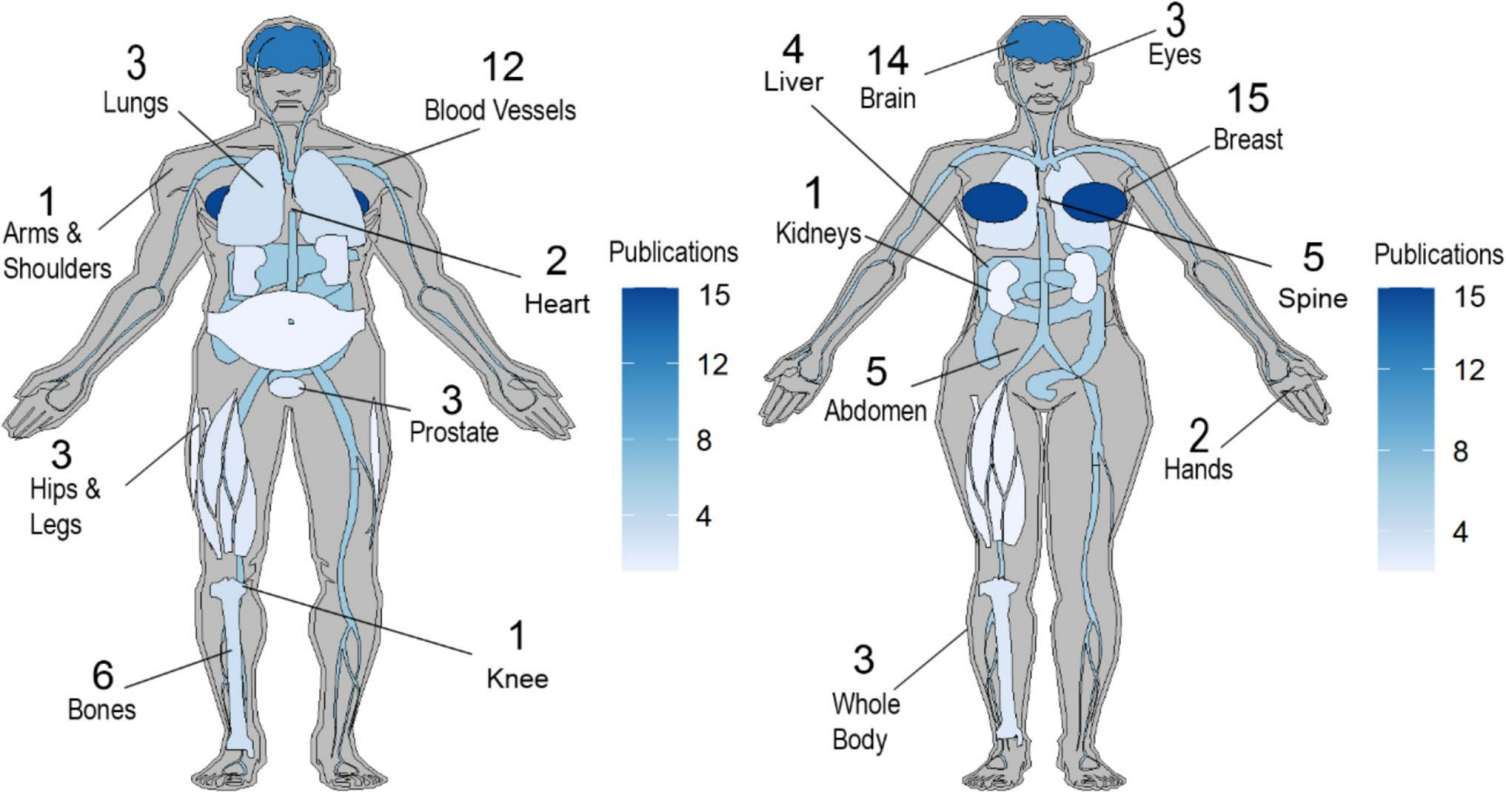
Distribution of anatomical regions investigated in included studies

A large proportion of studies (*n* = 32, 37%) utilized in vivo or ex vivo human data, while 17 studies (20%) used animal data and 60 (70%) used in silico or in vitro data. Several publications incorporated multiple types of data. Among all the studies, the anatomical regions varied widely, with some publications focusing on multiple anatomical regions. The breast was the most studied area (*n* = 15, 17%), followed by the brain (*n* = 14, 16%), blood vessels (*n* = 12, 14%), bone (*n* = 6, 7%), abdomen (*n* = 5, 6%), and spine (*n* = 5, 6%). Other regions studied included liver (*n* = 4, 5%), lungs (*n* = 3, 3%), eye (*n* = 3, 3%), prostate (*n* = 3, 3%), whole-body (*n* = 3, 3%), hand (*n* = 2, 2%), heart (*n* = 2, 2%), hip (*n* = 2, 2%), kidney (*n* = 1, 1%), knee (*n* = 1, 1%), legs (*n* = 1, 1%), shoulder (*n* = 1, 1%) (Fig. 4). Among the animal studies, most (*n* = 11, 13%) focused on mice or rats, followed by chickens (*n* = 2, 2%), fish (*n* = 1, 1%), insects (*n* = 1, 1%), rabbits (*n* = 1, 1%), and a diverse group of animals (*n* = 1, 1%).

#### Validation Procedure

All studies reported validation procedures. Internal validation was performed in 57 studies (66%), while only 4 studies (5%) conducted external validation. Furthermore, 25 studies (29%) developed methodologies without using training data, instead evaluating their performance on separate test sets.

### Ultrasound-Based Tomographic Imaging Modalities and Reconstruction Methods

**Fig. 5:**
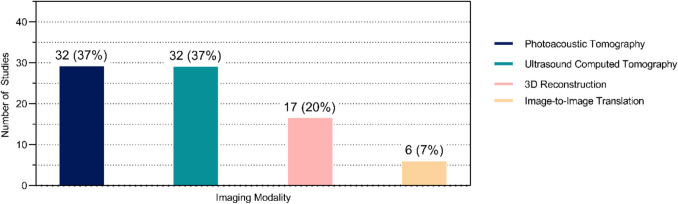
Bar chart illustrating the image modalities employed in the included studies

Four categories of ultrasound-based tomographic imaging modalities were identified: PAT (*n* = 31, 36%) [17, 23–52], USCT (*n* = 31, 36%) [16, 19, 53–81], 3D reconstruction (*n* = 17, 20%) [18, 82–97], and synthetic imaging (*n* = 6, 7%) [9, 10, 98–101] (Fig. 5). Additionally, one study (1%) focused on developing a hybrid dual-mode PAT/USCT imaging system [102].

**Fig. 6:**
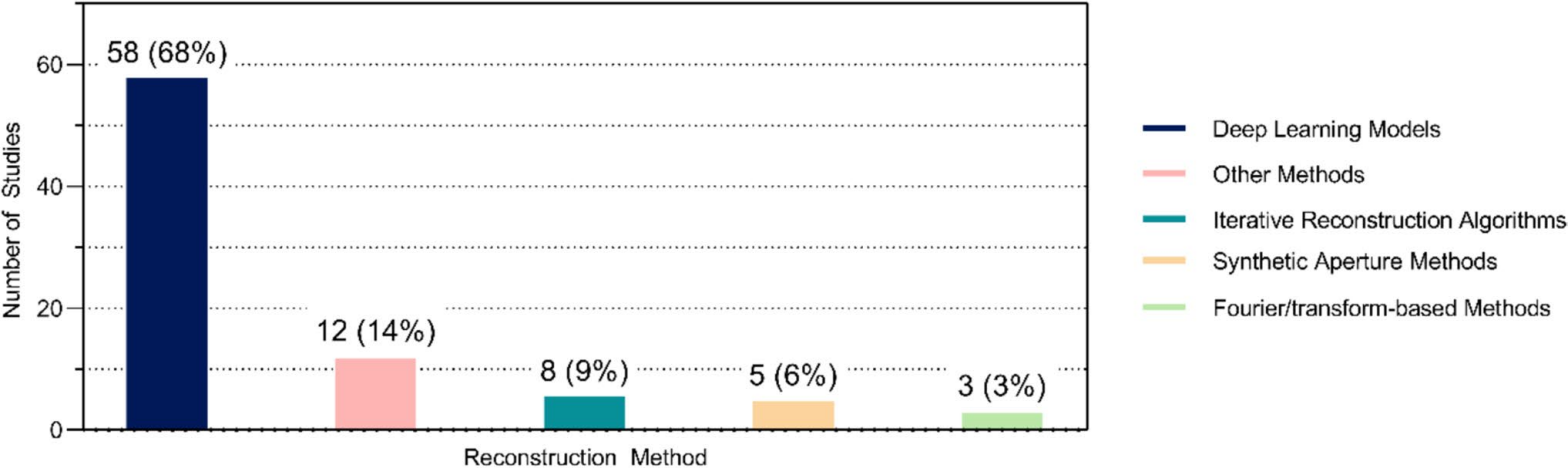
Bar chart summarizing the categories of reconstruction methods used in the included studies

To reconstruct and generate these imaging modalities, various methods were utilized. Deep learning-based algorithms were the most prevalent (*n* = 58, 67%) [9, 10, 16–18, 28–31, 33, 34, 36, 37, 41–49, 51, 62, 65, 67, 68, 70, 72, 73, 75–81, 90, 91, 93, 95–97, 100, 101, 103–105] [26, 27, 53, 54, 85–88, 98, 99], followed by iterative reconstruction algorithms (*n* = 8, 9%) [19, 32, 35, 40, 57, 59, 74, 106], synthetic aperture methods (*n* = 5, 6%) [58, 60, 61, 64, 102], Fourier/transform-based methods (*n* = 3, 3%) [23, 55, 69], and other miscellaneous approaches (*n* = 12, 14%) [24, 25, 39, 56, 63, 66, 71, 82–84, 89, 92] (Fig. 6). Among the deep learning methods, CNNs and U-Net architectures were most common (*n* = 28, 32%) [9, 17, 18, 26, 28, 30, 31, 33, 36, 43, 44, 47, 48, 53, 54, 65, 67, 70, 73, 76, 77, 79–81, 90, 95, 96, 104]. These were followed by hybrid methods combining deep learning techniques with classical reconstruction techniques (*n* = 10, 11%) [37, 38, 45, 49, 51, 72, 75, 88, 91, 93], GANs (*n* = 8, 9%) [10, 29, 52, 62, 68, 99–101], model-based or physics-informed architectures (*n* = 8, 9%) [16, 27, 34, 41, 42, 46, 78, 97], diffusion models (*n* = 2, 2%) [85, 98], and neural implicit models (*n* = 2, 2%) [86, 87]. Among the other miscellaneous approaches, studies included deconvolution algorithms, edge-preserving sound-speed reconstruction methods, adaptive kernel regression methods, and full-waveform inversion algorithms, among others. From this point onward, percentages are calculated relative to the total sample size of the respective category they reference.

#### Photoacoustic Imaging

Deep learning methodologies dominated the reconstruction methods for PAT imaging, appearing in 23 studies (74%). This was followed by iterative reconstruction algorithms (*n* = 4, 13%), other miscellaneous approaches (*n* = 3, 10%), and a Fourier/transform-based method (*n* = 1, 3%) (Table 1). Overall, the PAT studies encompassed a diverse range of anatomical regions, without any notable focus on a specific organ.

**Table 1:** Overview of reconstruction methods categorized by imaging modality

First author	Country	Year of publication	Primary objective	Reconstruction method	Sample sizes	Validation method	Dataset details
**Photoacoustic imaging**							
Boink et al	Netherlands	2018	Develop a modular framework for directional and higher-order reconstruction in PAT	Variational framework using TV, TGV, and directional wavelet regularization	n/a	Training-free validation	Synthetic and experimental vascular phantom datasets using alginate and iron oxide nanoparticles
Davoudi et al	Switzerland	2019	Develop a U-Net-based framework to restore sparse optoacoustic data	U-Net CNN trained to mitigate undersampling and limited-view artifacts	Synthetic: 100 phantoms; In vivo: 100 cross-sectional images per mouse for 6 mice (training: 420 training, validation: 120, testing: 60)	Internal validation	Synthetic: phantom data of paper-printed absorbing targets in agar cylinder; in vivo: cross-sectional images of mouse body
Guan et al	USA	2020	Develop a dense U-Net framework (Pixel-DL) for limited-view PAT	Fully Dense U-Net for artifact correction and image enhancement	Simulated training data: 500 synthetic vascular images; Testing: 50 mouse brain vasculature images Fundus: Training: 15 images (500 with data augmentation); Testing: 30 images (50 with data augmentation). Lung: Training: 15 low-dose CT scans (500 with data augmentation); Testing: 35 images (50 with data augmentation)	Internal validation	Synthetic vasculature phantoms generated with MATLAB; in vivo mouse brain datasets derived from micro-CT images; High-Resolution Fundus Image Database; ELCAP Public Lung Image Database
Lan et al	China	2022	Combine CNN and variational methods for limited-view PAT	Deep learning variational gradient descent with adaptive regularization	Simulated data (vessel phantoms): 3600 training images, 400 testing images. In vivo data (mice abdomen): 629 training images, 70 testing images	Internal validation	Simulated vessel-like phantoms created using k-Wave toolbox; in vivo data from PA scans of mice's abdomen with a 180° transducer array
Antholzer et al	Austria	2018	Combine FBP with U-Net for sparse PAT	Two-step framework: FBP preprocessing and U-Net artifact correction	Training: 2000 training pairs (1000 without, 1000 with noise), testing: 50 pairs	Internal validation	Synthetic ellipse phantoms generated on a 128 × 128 grid
Lan et al	China	2020	Develop a hybrid Y-Net for limited-view PAT	Y-Net: dual encoders for raw signals and beamformed images	Synthetic vessel-like phantoms: Training: 4700 sets, testing: 400 sets; one chicken breast tissue; one human palm	Internal validation	Synthetic data generated via k-Wave MATLAB toolbox with noise; in vitro data from chicken breast phantom; in vivo data from human palm scans with linear array probe
Köstli et al	Switzerland	2001	Develop a Fourier-based reconstruction method for 3D optoacoustic tomography	Fourier decomposition to reconstruct pressure distributions	n/a	Training-free validation	Gelatin phantoms mimicking blood vessels in scattering media and body of a small insect
Lu et al	China	2020	Use GANs for high-quality limited-view PAT	LV-GAN: GAN with U-Net-like generator and MSE/adversarial losses	Simulated data: Training: 10,368 pairs of limited-view and full-view; validation: 1296 pairs; testing: 1296 pairs. Experimental data: training: 13,392 pairs of limited-view and full-view; validation: 1674 pairs; testing: 1674 pairs. Kidney, number n/a	Internal validation	Simulated vessels and experimental kidney phantoms imaged at multiple angles
Rajendran et al	Singapore	2021	Enhance PAT reconstruction without radius calibration	Dense U-Net with Conv-Long Short-Term Memory (LSTM)	1260 PAT images (1000 simulated and 260 in vivo). training: 1133 images, validation: 63 images, testing: 63 images	Internal validation	Simulated datasets generated using numerical phantoms (vessel and point target shapes); in vivo data from Sprague Dawley rats
Deán-Ben et al	Germany	2012	Develop a model-based method to improve resolution and contrast	Model-based least squares inversion with Tikhonov regularization	n/a	Training-free validation	Simulation: single absorber; Experimental: mouse heart in agar phantom
Lan et al	China	2021	Build a real-time PAT system with LSTM-based reconstruction	LSTM encoder-decoder for reconstructing PA signals	Simulated data: training: 4500 samples, testing: 1000 samples; In vivo fish experiment: training: 1500 samples, testing: 400 samples	Internal validation	Synthetic disc data from MATLAB toolbox k-Wave; in vivo PA imaging data acquired from fish using a 120-channel transducer array
Choi et al	South Korea	2022	Improve volumetric PAT using 3D U-Net	3D progressive U-Net	Training: 1089 single-volume datasets, 1069 volumes from whole-body scanning of 18 rats, 10 kidney-specific volumes, and 10 brain-specific volumes Testing: tumor-bearing mice and human palms	External validation	Full-body imaging of 18 rats, kidney-specific volumes, brain-specific volumes, 3 or 4-week-old Sprague Dawley rats with tumors, human palm of one healthy volunteer
Hakimnejad et al	Iran	2024	Develop DensePANet GAN for sparse PAT reconstruction	FD-UNet + + for end-to-end GAN-based post-processing	- Simulated vessels dataset: training: 1600 images, testing: 400 images - Mouse-Abdomen: training: 4000 images; testing: 1000 images; - Brain Tumor MRI: training: 1057 images; testing: 264 images	Internal validation	Simulated Vessels: Synthetic PAT dataset created using MATLAB toolbox k-Wave, augmented to 2000 images Mouse-Abdomen: Whole-body mouse dataset with 256 tomographic projections, augmented to 5000 images Brain Tumor MRI: Public dataset of 1321 images from Kaggle, converted to PAT modality using K-Wave Toolbox
Li et al	China	2021	Use deep learning for artifact correction in PAT	CNN-based iterative optimization	Training: 4000 images, testing: 400 images	Internal validation	4400 photoacoustic images simulated using the public dataset DRIVE (digital retinal images for vessel extraction)
Zhang et al	China	2023	Develop a deep learning-based U-Net framework to correct skull-induced acoustic aberrations and improve photoacoustic tomography imaging of cerebral arteries	U-Net, followed by delay-and-sum reconstruction	Training: 120 PA sonograms, testing: 60 PA sonograms	Internal validation	Brain numerical phantoms based on 180 MRA and T1 MRI from IXI-dataset
Antholzer et al	Austria	2018	Develop efficient PAT reconstruction algorithms using deep learning to address undersampling artifacts	Combine FBP with U-Net and S-Net CNNs	Training: 1000 images, testing: 200 images	Internal validation	1200 synthetic ring-shaped phantoms with random variations
Bardsley et al	USA	2018	Develop methods for quantitative two-photon PAT to separate single-photon and two-photon absorption	Two-step reconstruction combining pressure recovery and nonlinear absorption modeling	n/a	Training-free validation	Synthetic phantoms with noise (0–10%)
Lan et al	China	2019	Develop a framework for PAT reconstruction using multi-frequency ultrasound sensor data	Modified U-Net architecture (DU-Net)	Training: 4000 datasets with numerical phantom and vessels’ PA signals Testing: n/a	Internal validation	Numerical phantoms and segmented vessels from CT imaging
Susmelj et al	Switzerland	2024	Improve limited-view PAT by addressing domain gaps between simulated and experimental datasets	Single domain adaptation network	Simulated: Training: 5400 frames, testing: 50 frames, validation: 50 frames; Experimental: Training: 5501 frames, testing: 32 frames, validation: 32 frames;	Internal validation	Simulated vessel-like phantoms with realistic boundary variations Experimental forearm data acquired with a linear and multi-segment transducer
Shan et al	USA	2019	Develop SR-Net for simultaneous sound speed and pressure reconstruction in PAT	SR-Net: deep learning with gradient-based updates	Training: 5120 images, training: 1024 images	Internal validation	Synthetic Shepp-Logan phantoms with varying sound speeds
Tong et al	China	2020	Develop FPnet for limited-view PAT domain transformation integrated with U-Net post-processing	FPnet for domain transformation with U-Net refinement	- Brain: training: 22,211 MRI images, validation: 267 images, testing: 276 images; - Abdomen: training: 8273 MRI images, validation: 368 images; testing: 336 images, - Vessel: training: 4′000 images, validation: 200 images, testing: 200 images; - LiverCancer: testing: 601 MRI images; - MSOT-Brain: training: 698 PAT images, testing: 64 images; - MSOT-Abdomen: training: 575 PAT images, testing: 124 images	Internal validation	Brain dataset from The Cancer Imaging Archive; Abdomen and LiverCancer datasets from First Affiliated Hospital, Jinan University; Vessel dataset obtained by randomly cropping images from DRIVE dataset; MSOT-Brain and MSOT-Abdomen contain PAT images of nude mice from MSOT inVision 128 system
Poudel et al	USA	2020	Develop an optimization-based algorithm for transcranial PAT to address skull aberrations	Iterative reconstruction using finite-difference time-domain approach and optimization-based reconstruction method	n/a	Training-free validation	Simulations: Skull phantom with superficial and cortical vessel structures; Experiments: Acrylic skull phantom with painted cortical vessels
Hauptmann et al	United Kingdom	2018	Combine model- and learning-based approaches for limited-view PAT	Deep gradient descent using CNNs for iterative reconstruction	Simulated: training: 1024 volumes, testing: 16 samples In vivo: number n/a	Internal validation	Lung vessel phantoms segmented from the ELCAP Public Lung Image Database; in vivo data from human palm acquired via Fabry–Perot scanner with sub-sampling
Schwab et al	Austria	2019	Improve PAT reconstruction with truncated SVD and CNN regularization	Two-step approach: SVD preprocessing + CNN refinement	Training: 3500 pairs, testing: 500 pairs	Internal validation	Shepp-Logan phantoms with deformations and noise
Guan et al	USA	2021	Develop Dense Dilated U-Net (DD-UNet) for sparse and limited-view PAT	DD-UNet with dense connectivity and dilated convolutions	Training: 1000 phantoms for spheres, lung vasculature, and breast vasculature. Testing: 500 phantoms for each category	Internal validation	Spheres: synthetically generated; Lungs: ECLAP Public Lung Image Database; Breast: Synthetic phantoms generated using analytic approach
Wang et al	China	2004	Develop deconvolution-based reconstruction for PAT without impulse response	Modified filtered back-projection	n/a	Training-free validation	Cylindrical phantoms mimicking breast tumors
Zhang et al	China	2012	Develop TV-based gradient descent for sparse PAT reconstruction	Gradient descent algorithm with TV minimization constraints	n/a	Training-free validation	Shepp-Logan phantoms and experimental gelatin absorbers
DiSpirito III et al	USA	2021	Develop Fully Dense U-Net (FD U-Net) for reconstructing undersampled photoacoustic microscopy	FD U-Net with dense blocks	Training: 304 images, validation: 39 images, testing: 38 images	Internal validation	In vivo mouse brain microvascular images
Paul et al	USA	2025	Introduce and evaluate a lightweight model-GE-CNN architecture to improve PAT reconstructions for low-element linear array	Lightweight dual-path CNN architecture combining noisy initial reconstructed image and gradient of data fidelity	Training: 320 PA image pairs Validation: 40 PA image pairs Testing: 40 PA image pairs	Internal validation	256-element sub-pitch dense PA dataset of numerical simulations, including circular phantom images, numerical blood vessels, and retina blood vessels In vivo rat liver imaging data
Chinnaiyan et al	India	2025	Develop a hybrid fuzzy CNN–BiLSTM with particle swarm optimization to improve PAI reconstruction	Modified fuzzy CNN-BiLSTM architecture	Synthetic experiments: Training: 5000 images Validation: 3000 images 9 Numerical human brain phantoms	Internal validation	3D brain numerical phantoms with vasculature from IXI dataset
Liu et al	China	2025	Develop a qPAT algorithm that separates light fluence from absorption coefficient (AC) to refine AC imaging accuracy	Kernel singular value decomposition algorithm employing orthogonal matching pursuit (OMP)	PA-US image pairs of 3 ink-filled tubes in agar/Intralipid; In vivo: PA-US image pairs of 1 mouse hind-limb	Training-free validation	Phantom: 3 ink-filled tubes in agar/Intralipid In vivo: 1 mouse hind-limb
**Ultrasound tomography**							
Linger et al	France	2023	Develop high-quality 3D dual-mode PA/US imaging with precise co-registration	Delay-and-sum beamforming optimized using thread phantoms	n/a	Training-free validation	Simulated: wire and gel phantoms; Experimental: thread and dual-contrast phantoms
Jiřík et al	Czech Republic	2009	Introduce and evaluate regularized image reconstruction techniques for ultrasound transmission tomography, focusing on sound-speed maps for breast cancer diagnostics	Edge-preserving regularized algebraic reconstruction	Synthetic dataset: Simulation using a 64 × 64 × 8 voxel volume; Breast phantom	Training-free validation	Synthetic datasets: Simulated 3D breast tissue model with Gaussian noise; Phantom data: Experimental data from a 3D USCT prototype on a breast phantom
Simonetti et al	UK	2009	Introduce a 3D Synthetic Aperture Diffraction Tomography (SADT) method for breast imaging using toroidal ultrasound arrays	Synthetic Aperture Beamforming (SABF) followed by deconvolution with point spread function	Numerical simulations: Sphere phantom with 80 transducers at 79 vertical positions	Training-free validation	Synthetic data: Homogeneous fluid sphere immersed in water, sound speed 1485 m/s (sphere) vs. 1500 m/s (water)
Ruiter et al	Germany	2008	Evaluate the feasibility of a 3D USCT system for breast cancer diagnosis	Synthetic Aperture Focusing Technique (SAFT) for reflectivity imaging; Feldkamp-Davis-Kress algorithm for speed of sound and absorption	n/a	Training-free validation	Experimental data: clinical breast phantoms and gelatine phantoms (e.g., nylon threads, mimicking lesions)
Fan et al	Germany	2021	Develop a memory-efficient neural network architecture for reconstructing USCT images directly from the frequency domain to the image domain	CNN with down-up sampling blocks	Training: 76,000 simulated data, validation: 2000 simulated data, testing: 2′000 OA-breast data	External validation	ImageNet dataset; OA-Breast dataset includes realistic breast tissue models
Gao et al	USA	2023	Introduce an innovative deep learning-based solution for USCT's	ConvNeXt block concept (evolution of CNN)	Training: 16,000 data points, testing: 4000 data points	Internal validation	Simulated data comprising sequences of randomly generated circles with eight distinct SoS values (simulated by K-wave toolxbox)
Long et al	China	2023	To achieve high-quality speed of sound reconstruction in USCT under sparse sampling conditions using a convolutional neural network	Bent-ray algorithm followed by CNN (SRSS-Net)	- Synthetic Ellipse Dataset: exclusively used for testing - Synthetic Breast Phantom Dataset: training: 703 images; testing: 189 images - Experimental Gelatin Phantom Dataset: training: 200 images, testing: 40 images - Ex Vivo Animal Organ Dataset: training: 300 images, testing: 42 images - Inhomogeneous Breast Phantom with Lesions: training: 90 images; testing: 10 images	Internal validation	Synthetic Ellipse Dataset: 2000 images of circles and ellipses of different sizes and intensities, exclusively used for testing Synthetic Breast Phantom Dataset: 892 slices of patient breast volumes Experimental Gelatin Phantom Dataset: 240 images of gelatin phantom immersed in water tank Ex Vivo Animal Organ Dataset: 342 images of ex vivo animal organs Inhomogeneous Breast Phantom with Lesions: 100 images of inhomogeneous breast phantoms with lesions
Ren et al	China	2024	To develop a reliable quantitative imaging framework for brain tissues using a physics-embedded neural network (PEN-FWI) with deep learning-based full waveform inversion	Physics-embedded neural network with deep learning-based waveform inversion (PEN-FWI), consisting of two components: forward CNN and inversion sub-neural network	Training: 2056 samples (offline testing); 1024 samples (online testing), validation: 256 samples, testing: 100 independent samples	External validation	1280 samples of ultrasound tomography data with 64 sources and receivers around a resin and silicone phantom to simulate skull and soft tissue
Zhou et al	China	2023	Test the efficiency and stability of a DL-UCT method for cortical bone imaging	End-to-end CNN	Training: 3200, testing: 800	Internal Validation	Bone models from in vivo X-ray CT scans of human tibia-fibula bone
Qu et al	China	2022	Develop a deep-learning-based approach for ultrasound sound-speed tomography reconstruction using Tikhonov pseudo-inverse priori	A hybrid approach combining a linear mapping module with Tikhonov pseudo-inverse and a non-linear mapping module using U-Net, forming the UT-Net and TU-Net architectures	Training: 855 samples, testing: five-cross validation with 171 samples in each group	Internal Validation	Numerical simulation data: 855 samples of simple geometrical models and complex breast models derived from breast MRI of 30 patients Prototype experimental data: circular phantom, breast phantom, small piece of chicken meat, and large piece of chicken meat
Prasad et al	USA	2022	Generate high contrast USCTs through deep learning and comparing it to the state-of-the-art waveform inversion method	CNN model directly inferring the SoS distribution from the recorded ultrasound data	Training: 19,000 pairs of ultrasound measurements and SoS models, testing: 2000; hyperparameter tuning: 1000	Internal validation	Synthetic dataset created by capturing synthetic phantoms consisting of several uniform circular disks mimicking tissue properties
Ruiter et al	Germany	2012	Demonstrate the first in vivo results using a 3D USCT prototype and compare volumes to MRI	3D SAFT employing voxel-wise reflectivity reconstruction with a matched filter	Testing: Over 14 million A-scans each for two volunteers collected with a semi-ellipsoidal aperture containing 628 emitters and 1413 receivers; compared with T1-weighted MRI slices	Training-free validation	Imaging of two healthy volunteers, with breast sizes ranging from A to D cup
Rymarczyk et al	Poland	2019	Present and evaluate an optimization-based image reconstruction algorithm for ultrasound transmission tomography	Optimization-based iterative reconstruction with linear inequality constraints	n/a	Training-free validation	Synthetic and real-world datasets with both single and multiple internal circular objects
Lyu et al	China	2020	Improve the accuracy of ultrasonic tomography image reconstruction using a multi-channel CNN method	Multi-channel CNN based on U-net	23,355 ultrasonic tomography samples; training, validation and testing split was mentioned, specific split n/a	Internal validation	Simulated ultrasonic tomography data
Lozenski et al	USA	2024	Develop a CNN-based learned FWI method with task-specific loss functions for speed-of-sound imaging in breast USCT	CNN (InversionNet) with task-informed loss and source encoding	Training: 1353 numerical breast phantoms (NBP); Testing: 41 NBPs	Internal validation	Numerical breast phantoms
Fan et al	Germany	2020	Introducing a DL-model (MI-Net) which combines Unet and DenseNet for USCT reconstruction in short time	MI-Net (combination of Unet and DenseNet), an end-to-end network reconstructing images directly from a frequency dependent pressure field	Training: 76′000 2 d simulated images, validation: 2000 simulated data, testing: 2000 images from KIT phantoms and OA-Breast	Internal validation	2D simulated images from OA-Breast model data for training; 2000 images from KIT phantoms and OA-Breast for testing
Fan et al	Germany	2021	To develop an accurate and fast image reconstruction algorithm for USCT using a hybrid model-data-driven approach	Dual-domain neural network architecture based on the primal–dual hybrid gradient method	Training: 8000 images, validation: 1000 images, testing: 1000 images	Internal validation	USCT Data: 4000 2D slices (of which 2′000 are augmented to 8′000 for training) extracted from OA-Breast dataset
Peterlik et al	Czech Republic	2008	Develop and evaluate a regularized image reconstruction method for ultrasound attenuation tomography to stabilize and improve the reconstruction of attenuation maps	Edge-preserving regularization with iterative half-quadratic optimization for algebraic reconstruction	n/a	Training-free validation	Simulated data of synthetic attenuation maps with three homogenous regions and sharp boundaries with 100 transducers and 7228 sender-receiver combinations
Jiřík et al	Czech Republic	2012	Develop and evaluate an edge-preserving sound-speed reconstruction method for 3D ultrasound transmission tomography using sparse apertures	Edge-preserving regularized algebraic reconstruction combined with synthetic aperture focusing	n/a	Training-free validation	Synthetic data modeled breast tissues; experimental data from a clinical breast phantom using 3D USCT
Quan et Huang	USA	2007	Investigate the capability of time-of-flight transmission ultrasound tomography with a ring array for reconstructing sound-speed images of the breast	Time-of-flight tomography with bent-ray inversion, solving the eikonal equation using a finite-difference method	n/a	Training-free validation	Simulated data from breast phantoms with varying shapes and sizes, including tumors
Long et al	China	2024	To develop an efficient approach for direct speed-of-sound image reconstruction using an improved conditional GAN	Conditional Wasserstein GAN with spatial-channel attention blocks	- Synthetic circle dataset: training: 48′000 images, testing: 12,000 images - Normal breast phantom dataset: training: 3612 images, testing: 908 images - Breast cancer phantom dataset: training: 34,002 slices, testing: 8575 slices	Internal validation	Synthetic circle dataset: 60′000 images of randomly generated nonoverlapping uniform disks Normal breast phantom dataset: 4520 images from 3 healthy breasts Breast cancer phantom dataset: 42′577 CT slices of 150 patients with breast cancer
Simonetti et Huang	United Kingdom	2009	Develop a 3D diffraction tomography method using synthetic aperture principles for high-resolution imaging of complex object	Synthetic aperture beamforming followed by deconvolution for accurate sound-speed and shape reconstruction	n/a	Training-free validation	Simulations of a homogeneous sphere with a radius of 20 mm using 80 transducers across 79 positions
Bernard et al	France	2017	Develop a full-waveform inversion method for quantitative imaging of long bones, focusing on speed of sound and structural mapping	Full-waveform inversion using quasi-Newton methods	n/a	Training-free validation	Phantom cylinders; Simulations of 2D tibia-fibula bone pair, with 8 sources and 128 receivers
Soleimani et al	United Kingdom	2024	Develop a portable USCT system and compare ultrasound brain tomography generated by deep learning (CNN + LSTM) vs. deterministic methods	Combined CNN and Long Short-Term Memory (LSTM)	Training: 35,000 synthetic cases, validation: 4000 cases	Internal validation	Phantom of a physical model of the human head
Huang et al	USA	2007	Demonstrate reflection image reconstruction using the split-step Fourier propagator for ultrasonic breast imaging	Split-step Fourier propagator with inward wavefield continuation in frequency-space and frequency-wavenumber domains	n/a	Training-free validation	Synthetic data using a computer-generated breast phantom with 4096 transducers arranged in a ring of 20 cm diameter
Zhao et al.,	Germany	2020	Introduce an efficient fully learned image reconstruction method based on a CNN and compare it to three other neural networks and two traditional reconstruction algorithms	CNN, multiple W-net (mWnet_1 and mWnet_4)	Training: 47,998 images, validation: 2000 images, testing: four test phantoms, nine breast phantom images	External validation	49,998 natural grayscale images from Image Net, four standard test phantoms, nine medical images from OA-Breast phantom dataset
Chung-Jukko et al	United Kingdom	2024	Develop Virtual Extended-Range Ultrasound Imaging (VERT), an algorithm for high-resolution, extended-range bone imaging with virtual transducers around the ROI	VERT, which improves upon bent-ray tomography with virtual transducers placed on the region of interest	1 Smiley80 (300 virtual transducers), 1 Bone200 (400 virtual transducers)	Training-free validation	Smiley80: 2D bone-like smiley structure; Bone200: ellipsoidal bone model with cancellous and cortical bone
Tong et al	China	2022	Develop a decomposition descent learning-based full waveform inversion method to reconstruct 3D transcranial ultrasound images for brain tissue and clot imaging	Linear residual decomposition-based FWI with transducer domain decomposition and dimension reduction	Simulated data: Training: 900 training samples, Testing: 100 samples for 2D, training: 700 samples, testing: 50 samples for 3D Experimental data: training: 220 samples, testing: 164 samples	Internal validation	Simulated brain models with synthetic velocity maps; experimental data obtained using brain and clot phantoms with 512-channel transducer array
Wen et al	China	2018	Develop an adaptive kernel regression method for freehand 3D ultrasound reconstruction	Adaptive kernel regression based on local statistics with GPU acceleration	Simulated data: 48 slices, 36 slices, and 29 slices Abdomen: 102 B-scan slices Liver: 167 B-scan slices	Training-free validation	Simulated CT data: three datasets with sampling every 3, 4, and 5 slices; experimental: abdominal phantom, liver data captured on healthy human subject
Liu et al	China	2025	Reconstruct high-quality reflection USCT images from sparse transmissions with a deep learning framework based on a generative adversarial network	Conditional GAN (UCT-GAN) with UNet-based generator and bottleneck attention module (BAM)	Training: 2100 pairs from 35 patients Validation: 600 pairs from 10 patients Testing: 300 pairs from 5 patients	Internal validation	In-vivo human breast USCT images at different transmission levels acquired on UltraLucid system with 2048-element ring
Li et al	China	2025	Achieve high-resolution ultrasonic bone imaging via deep learning–based full-waveform inversion	CEDD-Unet (Dual-decoder U-Net with Efficient Multi-Scale Attention module and ConvLSTM modules	Human Leg Bones: Training: 944 slices Testing: 236 slices Mouse femur: Training: 1120 slices Testing: 280 slices	Training-free validation	in vivo X-ray CT scans of human lower limb bones; images reconstructed from micro-computed tomography of murine femoral specimens
Ammaiappan et al	China	2025	Propose and evaluate a spectral graph CNN (SGCNN) to improve UTT image reconstruction from limited/noisy transmission data	SGCNN using Laplacian spectral filters	Training: 21,151 samples Testing: 2352 samples	Internal validation	UTT database which simulates one to three circular inclusions of various radiuses and places
**3D reconstruction**							
Ren et al	China	2023	Develop a 3D brain imaging reconstruction framework	Brain Imaging Full Convolution Network (3D AI algorithm)	Training: 240 samples, validation: 81 samples, testing: 80 samples Experimental: 1 brain-clot phantom	Internal validation	401 samples of high-resolution brain models; 1 brain-clot phantom
Sewify et al	Australia	2024	To develop a protocol for creating a comprehensive mosaic of the shoulder	Manual registration and fusion of multiple 3D US volumes	53 3D US volumes	Training-free validation	53 3D images of a healthy subject's shoulder
Tang et al	USA	2021	To develop a method for 3D reconstruction of lumbar spine surfaces from non-invasive ultrasound images acquired in free-hand mode	Late fusion-based U-net CNN	Training: 8 US volumes, testing: 1 US volume	Internal validation	In vivo scans from 9 New Zealand White rabbits using a 38-mm linear array transducer; Additionally registration and validation with CT scans
Guo et al	USA	2020	Develop a deep learning-based framework (DCL-Net) for reconstructing 3D ultrasound volumes from sensorless freehand ultrasound scans	DCL-Net using 3D convolutional layers with embedded self-attention and case-wise correlation loss to estimate probe trajectory and reconstruct volumes	Training: 500 TRUS videos, validation: 70 videos, testing: 70 videos	Internal validation	Clinical TRUS videos acquired from prostate cancer interventions with electromagnetic tracking data used as ground truth for motion estimation
Annangi et al	India	2022	To develop a method for creating patient-specific 3D anatomical surface models from B-mode ultrasound images	Utilizes U-Net architecture for segmentation and boundary extraction of liver regions from ultrasound frames. Specifically, 3 deep CNN models are developed based on U-Net architecture: Liver segmentation network; Sub-surface point rejection network; interface detection network	Training: 350 liver images for liver segmentation network, 300 images for sub-surface point rejection network training, 150 images for interface detection network; testing: 70 liver images for segmentation training	Internal validation	Liver images acquired using a GE LE9 ultrasound scanner with an attached electromagnetic sensor by an expert
Hohlmann et al	Germany	2023	To develop a fully automatic and robust pipeline for generating knee bone models from 3D freehand ultrasound scans	Combines CNN and SSMs	Training: 4150 US images, testing: 415 US images	Internal validation	US: 4565 images of knee joints of 10 subjects, aged 26–38 SSM: 2 SSMs of tibia and femur, built from 414 anonymized bone surface datasets
Quan et al	China	2021	Introduce a medical image 3D reconstruction framework based on transfer learning and Structure-from-Motion	Recovering camera motion and structure from a series of 2D medical slices through CNNs and 3D image reconstruction through SFM based CNN's	10 sets of ultrasound videos with 2133 sampled images from those sets; 7 sets used as the training set and 3 sets used as the verification set	Internal validation	2133 images (cropped to 350 × 400 image size) sampled from 10 sets of ultrasound videos of the LIFTL’s ultrasound library
Leblanc et al	France	2022	Develop a deep learning-based stretched reconstruction method for femoral artery imaging from 2D freehand ultrasound frames	Mask R-CNN segmentation for in-plane alignment, CNN for out-of-plane motion estimation, and linear interpolation for volume generation	111 tracked US sequences on 18 healthy volunteers. training: 6 sequences (4 volunteers, 2073 frames), validation: 3 sequences (1 volunteer, 702 frames), testing: 3 sequences (1 volunteer, 1004 frames)	Internal validation	Femoral artery sequences spanning from thigh to knee, annotated for segmentation, with ground truth motion data from optical tracking
Zhang et al	China	2022	Develop a robotic-assisted ultrasound scanning system for lumbar puncture, enabling automatic imaging and 3D visualization for surgical path planning	Linear interpolation and ray-casting algorithm using visualization toolkit for 3D visualization	136 2D ultrasound images of a lumbar spine model	Training-free validation	Synthetic lumbar spine model submerged in hydrogel
Luo et al	China	2021	Develop an online learning framework for sensorless freehand 3D ultrasound reconstruction under complex scan sequences using SSL and ADL	Differentiable reconstruction approximation with ConvLSTM, SSL for context consistency, and ADL for anatomical shape priors	- DDH (developmental dysplasia of the hip) Dataset: 101 US volumes (training: 85, testing: 15); - Fetal Dataset: 78 US volumes (training: 65, testing: 13)	Internal validation	DDH dataset including 14 volunteers and fetal ultrasound dataset from 78 volunteers
Sun et al	Netherlands	2025	Introduce an automated robotic ultrasound scanning (ARUS) system that localizes targets, plans trajectories, segments muscle and bone, and reconstructs 3D musculoskeletal structures	RA-UNet segmentation followed by edge extraction and point-cloud & mesh reconstruction	Ultrasound images: 200 (augmented to 1600); Training: 1,280 images Testing: 320 images Robotic scans: 3 trials; force-control tests: 3 trials	Internal validation	Custom musculoskeletal phantom (“muscle” + ulna and radius)
Methari et al	Spain	2025	Assess feasibility of reconstructing 3D left atrium and appendage from sparse 2D ultrasound planes using deep learning	Two pipelines: E-Pix2Vox + + trained on sparse 2D TEE slices; Neural implicit function with shape prior trained on sparse axial, coronal, sagittal planes; both output 3D volumes/meshes	Training: 1086 meshes Validation: 180 meshes Testing: 24 meshes	Internal validation	Synthetic 2D planes generated from CT-based statistical shape model of atrial fibrillation patients
Chen et al	China	2024	Introduce FUNSR, a self-supervised neural implicit method that learns signed-distance functions from US volumes to reconstruct 3D volumes	FUNSR: self-supervised implicit SDF learning with query-point sampling around volumetric point clouds	Four datasets: Phantom dataset: 2 3D US scans CCA dataset: 10 shapes CAB dataset: 77 shapes Prostate dataset: TRUS volumes from 108 patients	Training-free validation	Four datasets: Phantom dataset: 2 hip joint phantoms CCA dataset: 10 shapes from 5 volunteers CAB dataset: 77 cases Prostate dataset: TRUS volumes from 108 patients from MICCAI 2023 MRI to US Registration for Prostate Challenge
Yu et al	China	2025	Generate patient-specific 3D cardiac “anatomical twins” from sparse multi-view 2D ultrasound	UltraTwin: coarse-to-fine Conditional Diffusion Transformer with multi-view feature fusion, cardiac template priors, and anisotropic refinement	891 patients total (96 ECG-gated strictly paired; 795 pseudo-paired). Multi-view reconstruction: 130 samples (train 96/val 10/test 24). Implicit autoencoder training: 1,379 CT cardiac models (454 Total Segmentator + 891 internal)	Internal validation	9 hospitals; US multi-view videos with 12 standard echo views paired with CT scans with < 10 days between examinations
Al-Zogbi et al	USA	2025	Demonstrate autonomous robotic US scanning and 3D reconstruction of bifurcated femoral arteries	Ellipse-fit per frame, followed by centroid back-projection, and pixel to 3D world coordinate transformation	Training: 3838 images Validation: 476 images Testing: 488 images	Internal validation	Two CIRS phantoms with custom hydrogel topologies derived from 5 patient CTs
Ungi et al	Canada	2020	Develop image segmentation and 3D volume reconstruction methods for scoliosis measurements	U-Net–based frame-wise bone segmentation followed by tracked-US volume reconstruction for 3D visualization	Training: 3290 adult US images Testing: 1,892 pediatric US images	Internal validation	Tracked US (MicrUs EXT-1H) of eight adult volunteers; Tracked US of six pediatric scoliotic patients
Effatparvar et al	Canada	2022	Assess and improve an ultrasound-based 3D bone modeling pipeline for lumbar spine (with posterior soft tissues)	Mechanized circumferential ultrasound acquisition, followed by classical image processing (intensity transforms, Laplacian, high-pass filtering, thresholding), and segmentation in Amira to generate a 3D surface model	6 US scans	Training-free validation	6 cadaveric lumbar spines (T12–S2) in home-made cylinder plastic frame filled with porcine gelatine. Images collected by computer-assisted mechanical frame for whole-length ultrasonography
**Synthetic imaging**							
Jiao et al	United Kingdom	2020	To develop and validate a self-supervised and trainable model that can synthesize MRI fetal brain images from US images	Deep learning framework, including convolutional layers, up-convolutional layers, pooling and EdgeNet	Training and validation: 28′800 US slices, 480 MRI slices; testing: 7′200 US slices, 120 MRI slices	Internal validation	107 US from a multi-centre, ethnically diverse dataset from normal pregnancies. 2 MRI volumes from fetal brain atlas and Hammersmith Hospital. Additional MR volumes were synthetically generated
Sun et al	China	2021	To investigate the feasibility of using a stacked GAN to synthesize pseudo-CT images based on US images	Stacked GAN involving a conditional GAN for low-resolution pseudo-CT synthesis and a super-resolution GAN for high-resolution reconstruction	Training: 75 US and CT images, testing: 10 US and CT images, validation (fivefold cross validation)	Internal validation	85 US and CT cases/volumes of preoperative cervical cancer patients under volumetric modulated arc therapy
Vukovic et al	Australia	2023	To develop and validate a deep learning approach to enable synthesis of MRI volumes from US volumes	CycleGAN with a U-net structure using ResNet 9 block backbone for the generator and PatchGAN for the discriminator	Training: 100 US volumes, 25 MRI slices, validation (fivefold cross validation): 100 US volumes, testing: 20 US volumes	Internal validation	Thoracic spine phantom submerged in water; 220 US volumes T9 (incl. partial views of T8 and T10); 1 MRI volume of whole thoracic spine (T1–T12)
Singh et al	India	2023	To develop a deep learning framework to synthesize 3D MRI volumes from 3D ultrasound images of the brain	Pix2Pix GAN with a U-Net generator and a patch discriminator for 3D image translation	Training: 23 paired US and MRI cases	Internal validation	Pre-operative MRI and ultrasound data from 23 patients with low-grade gliomas
Salmanpour et al	Canada	2025	Evaluate state-of-the-art 2D/3D image-to-image (I2I) networks for US → MRI synthesis in prostate cancer using radiomics, expert qualitative review, and outcome classification	Ten I2I models compared: 2D Pix2Pix (best), 2D-CycleGAN, 2D-DiscoGAN, 2D-GcGAN, 2D-DualGAN; 2D-ContourDiff; 3D-CycleGAN, 3D-Autoencoder, 3D-UNet; 3D-Med-DDPM	794 image pairs; Training: 596 pairs Validation: 79 pairs External Testing: 119 pairs	Internal validation	TCIA Prostate MRI–US Biopsy dataset; 3D TRUS and T2-weighted MRI with segmentations from 794 patients
Silverstein et al	Israel	2025	Translate fetal brain US images to pseudo-MRI to improve visualization using a diffusion-based approach	Dual Diffusion Imposed Correlation (DDIC): unpaired diffusion-model translation	US: Training: 329 images Testing: 36 images MRI: 251 images	Internal validation	US images from Automated measurement of fetal Head Circumference dataset; 3D T2-MRI data from CRL fetal brain atlas database and FeTA challenge dataset

*TV*: Total variation, *TGV*: Total generalized variation, *PAT*: Photoacoustic tomography, *CNN*: Convolutional neural network, *PA*: Photoacoustic, *FPB*: Filtered back projection, *GAN*: Generative adversarial network, *MRI*: Magnetic resonance imaging, *MRA*: Magnetic resonance angiography, *SVD*: Singular value decomposition, *LSTM*: Long short-term memory, *US*: Ultrasound, *USCT*: Ultrasound computed tomography, *DL*: Deep learning, *CT*: Computed tomography, *SoS*: Speed of sound, *SAFT*: Synthetic aperture focusing technique, *FWI*: Full-waveform inversion, *3D*: Three-dimensional, *2D*: Two-dimensional, *UTT*: Ultrasound transmission tomography, *TRUS*: Transrectal ultrasound, *SSM*: Statistical shape model, *SSL*: Self-supervised learning, *ADL*: Adversarial deep learning, *TEE*: Transesophageal echocardiography, *CCA*: Common carotid artery, *CAB*: Carotid artery bifurcation, *ECG*: Electrocardiography

#### Ultrasound Tomography

Reconstruction methodologies were varied but dominated by deep learning approaches (*n* = 17, 55%). Other notable methods included synthetic aperture techniques, other miscellaneous approaches, and iterative reconstruction algorithms (each *n* = 4, 13%), and Fourier/transform-based methods (*n* = 2, 6%) (Table 1). Anatomically, the breast was the most commonly studied region (*n* = 12, 60%), while the remaining studies followed the broader distribution described earlier.

#### 3D Reconstruction

Deep learning methodologies were the most prevalent reconstruction approach (*n* = 12, 71%), followed by a variety of other miscellaneous methods (*n* = 5, 29%). Among the deep learning methods, models from the CCN/U-Net family (*n* = 5, 42%) were implemented, followed by diffusion/transformer models (*n* = 3, 25%), hybrid models combining classical reconstruction methods with deep learning (*n* = 3, 25%), and one model-based/physics-informed architecture (*n* = 1, 8%) (Table 1). Anatomically, four studies (24%) focused on the spine, with the remaining studies demonstrating no specific focus on an anatomical area.

#### Synthetic Imaging

All six studies (100%) employed deep learning techniques, with four (67%) using GANs, one (17%) using a CNN, and one (17%) using a diffusion-based approach. Regarding anatomical regions, 3 studies (50%) focused on the brain, 2 studies (33%) on the lower abdomen, and 1 (17%) on the spine.

## Discussion

This scoping review aimed to identify the data types and methodologies used to generate 3D tomographic imaging from ultrasound-based data. The findings revealed a predominant focus on ultrasound computed tomography (USCT) and photoacoustic tomography (PAT), with 3D reconstruction and synthetic imaging emerging more recently. Deep learning approaches dominated reconstruction techniques, including GANs, CNNs, U-Net variants, and hybrid deep learning models. Despite increasing research since 2018, particularly in Asia and Europe, challenges remain in computational efficiency, validation, and lack of generalization. Future work should enhance algorithm robustness, broaden anatomical applications, and improve clinical scalability.

This review encompassed a broad spectrum of studies spanning multiple years and institutions. The majority of studies were published from 2018 onwards, reflecting a growing interest in the field, likely driven by advancements in computational power and deep learning methodologies [107], which were all published after 2018, with only nine non-deep learning methods being published in the same time period. These advancements have enabled the synthesis and prediction of complex tomographic imaging data from “simpler” ultrasound data, which was previously practically unachievable.

Geographically, it was notable that Asian institutions led the research landscape, followed by European and North American institutions. This trend can likely be attributed to significant investments in artificial intelligence research in China, which accounts for 81% of the publications in this review from Asia, as well as the availability of large-scale medical datasets [108, 109]. These factors create an optimal environment for training deep learning models and thus driving advancements in synthetic medical imaging.

Validation procedures revealed a significant gap, as while all included studies reported some form of validation, very few conducted external validation. Even among those who performed external validation, some used simulated rather than real images, which further limits the validation. This lack of external validation is likely because many of the algorithms described were in their early stages of development and had yet to be fully optimized. In such cases, focusing on internal validation may often a logical first step before progressing to external validation. Additionally, the lack of external validation may arise from limited access to diverse and standardized ultrasound volumes. This highlights a critical limitation: the majority of algorithms remain far from clinical integration. To bridge this gap, further development is essential, including comprehensive external validation and comparative benchmarking against state-of-the-art algorithms, to ensure clinical robustness and applicability.

While initially focusing on generative synthetic imaging, we identified four major ultrasound-based tomographic imaging modalities (PAT, USCT, 3D reconstruction, and synthetic imaging). Despite their differences, some serving purely as visual imaging techniques and others incorporating functional information, they all share the common goal of utilizing ultrasound data to produce tomography-like images. It is important to note that the classification of the reconstruction methods is not inherently mutually exclusive. Approaches may combine aspects of multiple categories. For example, deep learning models such as PEN-FWI incorporate iterative optimization routines. Our classification is based on the predominant method used for image reconstruction as described by the original authors.

### Photoacoustic Tomography

Among the 86 articles included in this review, PAT emerged as the primary ultrasound-based tomographic imaging modality. Over the past decade, PAT has been gaining interest due to its potential as a complementary functional imaging technique [110]. PAT enables rapid, high-resolution imaging, including real-time cross-sectional scans and full-breast scans within seconds [110]. Its optical contrast enables the visualization of anatomical, functional, molecular, and histological features, making it particularly promising for oncological screening, diagnosis, and therapy [11]. PAT is based on the photoacoustic effect, where electromagnetic radiation, typically in the visible to near-infrared (NIR) spectrum, irradiates tissue and is absorbed by specific chromophores, such as hemoglobin, melanin, or water [110]. The absorbed energy induces rapid thermal expansion, resulting in localized pressure increases. This pressure dissipates as acoustic waves, which are subsequently detected by ultrasound transducers [111]. This absorption-based contrast enables greater tissue differentiation because the variations in optical absorption between tissue types are often greater than differences in typical acoustic impedance [111].

The PAT image reconstruction process represents an ill-posed inverse problem, which involves reconstructing the internal source of acoustic waves from surface measurements, which can be challenging due to incomplete data, measurement noise, and wave diffraction effects [112]. Furthermore, achieving high-quality reconstructions often requires a large number of sensors surrounding the tissue to ensure adequate data coverage, which may be prohibitively expensive and logistically impractical in in vivo clinical settings [113]. Thus, sparse data acquisition may be a viable alternative; however, it frequently leads to streaking artifacts that degrade image quality and limit its diagnostic utility [114]. Typically, this was addressed by model-based iterative methods [35, 106]; however, deep learning has emerged as a powerful alternative to conventional iterative techniques, offering enhanced image quality and computational efficiency. Recent studies have demonstrated the effectiveness of U-Net-based architectures and their variants, such as Dense U-Net, 3D Progressive U-Net, and FD U-Net, for correcting artifacts, enhancing volumetric images, and mapping undersampled data to fully sampled counterparts [17, 29, 31, 44]. For instance, Davoudi et al. proposed a U-Net framework trained on high-quality in vivo mouse scans to restore image quality from sparse datasets [17]. Their method successfully removed streaking artifacts, outperforming conventional iterative reconstruction techniques while validating robust performance on biological datasets. Despite its success, the authors highlight variability in results when using different training datasets, suggesting that retraining is likely necessary to achieve optimal performance in other imaging contexts.

Hybrid methods that combine physics-based reconstruction techniques with deep learning post-processing represent another promising avenue [37, 45]. For example, frameworks integrating filtered back-projection (FBP) or truncated singular value decomposition (SVD) with convolutional neural networks (CNNs) have demonstrated improved reconstruction quality [37, 45]. In such approaches, an initial image generated by a linear reconstruction algorithm serves as input to a CNN, which then refines the image by removing artifacts caused by undersampling. Antholzer et al. [37] exemplified this two-step methodology by coupling FBP with a U-Net architecture, achieving comparable or superior image quality to iterative methods such as total variation minimization while reducing computational time.

Despite the dominance of deep learning in PAT reconstruction, iterative methods remain relevant for specialized applications. For instance, Poudel et al. developed an optimization-based reconstruction method (OBRM) for transcranial PAT, using the 3D elastic wave equation to correct skull-induced aberrations [106]. Their approach improved resolution and cortical visualization while demonstrating superior contrast and noise robustness over conventional techniques. However, OBRM is strongly dependent on accurate physical modeling, making it prone to errors when applied to real-world, imperfect data [106].

PAT still faces several challenges, such as limited-view artifacts or limited image depth due to light attenuation, all of which can lead to incomplete or distorted images [110, 115]. Unlike ultrasound tomography, PAT relies on both optical absorption and acoustic wave propagation, which makes it more complex and sensitive to factors like signal loss and tissue variations [110]. Recent studies have shown that deep learning, especially hybrid approaches that combine traditional physics-based methods with data-driven techniques, can effectively address these issues [37, 45]. These methods offer better image quality, reduce artifacts, and improve computational efficiency. However, further work is needed to ensure these models are reliable across different systems and clinical settings, with a strong focus on experimental validation and improving their ability to generalize to new data.

### Ultrasound Tomography

Closely following photoacoustic tomography, USCT emerged as the second most common ultrasound-based tomographic imaging modality identified in this review. USCT has been explored since the early 1970s, particularly for breast cancer detection, but it remains an emerging imaging technology with limited clinical adoption [60, 116]. The focus of USCT on breast imaging in most of its applications can be attributed to two key factors. First, the widespread implementation of breast cancer screening programs may create a strong demand for non-invasive imaging methods to evaluate complex breast tissue [117]. Second, traditional ultrasound struggles with wavelength limitations when imaging dense or heterogeneous breast tissue, while the alternative, CT scanning, exposes patients to high levels of ionizing radiation [60]. USCT typically employs an array of transducers, which act as both transmitters and receivers, positioned around the region of interest [118]. This arrangement enables a full 360-degree acquisition of acoustic data, which has been applied not only in breast imaging but also in full-body [119] and brain USCT [16].

Reconstruction of the captured acoustical data, such as the speed of sound (SoS), is commonly performed using full waveform inversion (FWI) algorithms. FWI solves the wave equation by simulating the wave propagation process (i.e., a simulation of how the wave travels through the material), enabling the reconstruction of images with higher spatial resolution [81]. However, traditional FWI methods are prone to artifacts, computationally intensive, and unclear tissue boundaries, which limit their clinical utility [67, 81, 120, 121]. In this review, four of the 31 studies addressed such algorithms, while synthetic aperture focusing techniques (SAFT) were also explored as a reconstruction strategy. For example, Ruiter et al. [64] presented 3D USCT as a novel modality for breast cancer detection, integrating SAFT to achieve isotropic, high-resolution volumetric imaging. SAFT enhances resolution and reduces hardware complexity by combining signals from multiple transducer positions to simulate a larger effective aperture [122]. Their study demonstrated in vivo imaging of two healthy volunteers, comparing USCT results with MRI. The device successfully delineated breast surfaces and internal structures with high reproducibility. However, limitations such as sound-speed variations affecting resolution were noted, indicating areas for further improvement.

Deep learning emerged as the dominant reconstruction methodology (55%) in USCT, with all 17 deep learning studies published from 2020 onwards. The specific deep learning approaches varied substantially, with CNNs being the most prevalent. For example, Fan et al. developed a memory-efficient neural network architecture for USCT reconstruction directly from the frequency domain (i.e., analyzing the ultrasound waves based on their frequency rather than position or time) [78]. The model was trained on 2000 2D ultrasound breast slices and tested and internally validated on separate sets of 1000 images, respectively, from the same dataset. The authors were able to show that high-quality USCT reconstruction can be achieved through deep learning methodologies while even reducing computational duration. However, the authors highlight two common limitations of deep learning: the need for large amounts of training data and the lack of a guarantee that the model will achieve a global solution (i.e., one that closely matches the reference solution).

Hybrid methods, such as physics-embedded neural networks, offer a promising balance between physical interpretability and computational efficiency. These methods typically integrate a physical model, like speed-of-sound modeling, into a fully connected neural network. For instance, Ren et al. developed a physics-embedded neural network (PEN-FWI) to improve FWI for ultrasonic brain imaging, addressing skull-induced distortions [16]. This approach demonstrated robustness under noisy and limited data conditions, highlighting the potential of hybrid methods.

It is important to note that USCT faces challenges similar to those of PAT, particularly in image reconstruction. While PAT benefits from optical contrast, USCT relies solely on acoustic properties like sound speed and attenuation, making it highly sensitive to tissue inhomogeneities and acoustic aberrations [11, 123].

In summary, ultrasound tomography remains a promising but emerging imaging modality, particularly for breast imaging. Advances in deep learning, especially hybrid frameworks that combine physics-driven methods with data-driven algorithms, seem to have improved reconstruction quality and computational efficiency. However, overcoming the inherent challenges of USCT, such as tissue inhomogeneity, acoustic aberrations, and non-linear reconstruction problems, remains critical for broader clinical adoption.

### 3D Reconstruction

The development of 3D ultrasound (3D US) has expanded clinical applications of US by providing richer spatial information. However, 3D US is limited by a small field of view, making it challenging to obtain comprehensive imaging [97]. Therefore, reconstructing 3D volumes from multiple 2D ultrasound frames holds significant clinical potential [12]. Despite its promise, 3D reconstruction from 2D ultrasound data is highly complex due to in-plane and out-of-plane probe movements, which introduce shifts and misalignments. Traditional (non-deep learning) methods have been attempted, but these approaches have seen little clinical application due to their natural limitations [124]. One notable non-deep learning study captured in this review is a study by Zhang et al., which proposed a robotic-assisted ultrasound scanning system for lumbar spine imaging to aid lumbar puncture surgeries [92]. The system acquired 136 2D ultrasound images of a lumbar spine model and reconstructed them into a 3D volume using a ray-casting algorithm, a volume-rendering method that calculates light interactions along a path through the data. This method successfully identified anatomical landmarks, such as the spinous process gaps. However, the reconstructed images were partially defective due to ultrasound propagation limitations (e.g., issues such as signal loss, distortion, and artifacts), highlighting the challenges non-deep learning methods face in achieving high-quality reconstructions.

With the rapid advancements in deep learning, there may be growing potential for real-time 3D volume reconstruction from 2D ultrasound data. Recent literature has increasingly focused on deep learning-based methods for 3D reconstruction, with ca. 70% of the methods captured in this review relying on deep learning. For instance, Hohlmann et al. [91] presented a fully automated workflow for generating 3D knee bone models using ultrasound data. Their method combined a CNN for real-time segmentation of bone surfaces with a statistical shape model (SSM) to complete partial scans into full bone reconstructions. The system, which uses freehand 3D ultrasound with optical tracking, achieved submillimeter accuracy compared to MRI references, demonstrating its potential as a non-ionizing alternative to conventional tomographic imaging for surgical planning in knee arthroplasty.

A promising direction in 3D US reconstruction is bone modeling facilitated by segmentation. Ungi et al. demonstrated this by scanning the spines of volunteers with US transducers, after which a physician manually labeled the bone contours. A U-Net architecture was then trained to automatically segment the bones. A U-Net architecture was then trained to automatically segment the bones. The resulting segmentation maps were reconstructed into 3D volumes using position tracking information recorded for each US frame [83]. The authors further evaluated their approach in a dataset of pediatric patients with scoliosis, where they demonstrated that accurate spinal measurements were feasible.

Another approach is the method described by Effatparvar et al., which used cadaveric spine specimens stabilized in computer-assisted mechanical frames, which enabled whole-length US acquisition. The transducer was moved along the spine to acquire images of the entire specimen. These images were then compiled into a 360-degree DICOM dataset. Subsequently, a software segmentation tool was used to segment the spine to create 3D models [82]. A comparison with CT-based reconstructions showed comparable results. Both these forms of segmentation-driven 3D reconstruction approaches are increasingly well-established and show promise for clinical application in bone imaging.

Unique limitations for 3D volumetric reconstruction from 2D ultrasound data include probe movement inconsistencies, which can cause misaligned frames, limited field of view, leading to incomplete coverage, artifacts from acoustic shadows and noise, and the lack of depth information, requiring robust algorithms to infer spatial relationships accurately [18, 95, 104]. Additionally, variations in operator skill and anatomy further complicate achieving accurate, reproducible reconstructions [124]. Significant technical challenges remain, particularly for freehand ultrasound systems, which introduce inconsistencies in probe movement and frame alignment compared to more controlled setups such as robotic or fixed ultrasound systems [124].

In this context, tracking of the ultrasound probe relative to the patient has become crucial for 3D US reconstruction [125]. Rather than relying on patient breath-holding to minimize motion artifacts, tracking systems can monitor the movement of the region of interest during scanning [126]. This enables precise localization of the slices within a common 3D coordinate system using a range of sensors, including acoustic or optical sensors [127]. Alternative methods include mounting low-cost cameras on the probe to capture surface skin features, which can then be processed by localization algorithms to achieve accurate spatial reconstruction [128]. Regardless of the chosen method, robust probe localization is crucial for any form of freehand 3D ultrasound imaging [124].[123–125]

### Synthetic Imaging

Synthetic imaging aims to convert images between modalities, such as x-ray to CT, CT to MRI, MRI to CT, or ultrasound to CT, addressing limitations of tomographic imaging such as high radiation exposure and high costs [129, 130]. Leveraging ultrasound’s low-cost, non-invasive nature to produce high-quality tomographic images could greatly enhance clinical practice.

Our review identified six studies on ultrasound-to-tomographic synthetic imaging, all published from 2020 onward. All studies employed deep learning algorithms, which have only recently made such direct synthetic imaging feasible with the advent of GANs and CNNs. Among the synthetic imaging studies, four used GANs, while one employed a hybrid GAN-CNN architecture and another one a diffusion model.

For instance, Vukovic et al. [100] developed a CycleGAN-based method for real-time synthesis of MRI from US images, focusing on the T9 vertebrae of a thoracic spine phantom. This approach achieved spatial alignment between US and MRI volumes, validated with Dice and overlap metrics, and was confirmed anatomically valid by clinical experts in 80% of cases, highlighting the potential for real-time, tracking-free image generation.

A key advantage of CycleGANs is their ability to overcome the limitation of requiring paired datasets, which is typically a major challenge in synthetic imaging [131]. By learning mappings between input and output images without aligned training pairs, CycleGANs enable models to work effectively with unpaired data, significantly broadening their applicability [131].

Jiao et al. proposed a self-supervised method for synthesizing MRI-like images from unpaired fetal ultrasound data [9]. By assuming a shared anatomical latent space between US and MRI, their framework generates anatomically consistent MR images. This method outperformed autoencoders, GANs, and CycleGANs in qualitative and quantitative metrics such as mean opinion scores and deformation metrics [9]. Furthermore, it also addresses a long-standing challenge in ultrasound: recognizing anatomical structures. Traditionally, this requires labor-intensive annotation of ultrasound data. Their algorithm circumvents this by allowing anatomical structures to be annotated on the MRI data, which can then be transferred to the ultrasound domain [9]. However, the current research has relied on pre-processed ultrasound data (cropped and aligned), rather than full, unprocessed scans, which limits generalizability [9].

Another limitation lies in the fact that most of the studies included in this review focused on physiological datasets rather than pathological ones, meaning these models may not perform optimally in real clinical scenarios. Despite these challenges, synthetic imaging for ultrasound-to-tomographic imaging holds significant promise. While the number of studies in this field is currently small, it is likely to grow rapidly, driven by advancements in deep learning and the increasing focus on overcoming the limitations of traditional ultrasound imaging.

Although this review separates photoacoustic tomography, ultrasound computed tomography, 3D reconstruction, and synthetic imaging into distinct categories, these approaches share the common objective of reconstructing tomographic images from ultrasound data. Each modality is unique, with its own strengths and limitations. They also face similar challenges, including limited data availability, imaging artifacts, and high computational demands. At the same time, they differ fundamentally in their inputs, outputs, and hardware requirements. Therefore, it is important to recognize them as distinct categories while acknowledging that, although grouped under the same umbrella, opportunities for transferring knowledge between methods exist but remain limited.

### Deep Learning Methods

Deep learning algorithms are data-driven approaches that automatically learn patterns from input and output pairs (in this case ultrasound and corresponding tomographic imaging) using artificial neural networks [132]. These networks consist of layers of interconnected nodes, of which each performs a mathematical operation on input data[129]. The network is then trained to adjust the weights in the network based on the difference between its predictions and the actual ground truth, gradually improving its performance [133]. Early deep learning methods in medical image reconstruction were dominated by CNNs, which use spatial filters to extract hierarchical features from images [132]. Subsequently, GANs emerged, which typically employ a generator–discriminator architecture in which one network generates synthetic images while the other attempts to distinguish them from real ones [134]. More recently, diffusion models have emerged, which offer high-quality image synthesis by learning to reverse a noise-adding process and then reconstruct detailed outputs [135]. Finally, transformer architectures, which can learn relationships across the entire image instead of small local areas, are increasingly being explored [136].

Among all the methodologies identified for the generation or reconstruction of ultrasound-based imaging, deep learning methods emerged as the most common and promising approaches. These methods have demonstrated the ability to improve image quality, accelerate reconstruction times, and enable novel applications that were previously infeasible [17, 29, 67, 90, 100]. Nonetheless, important limitations remain.

One significant challenge is the lack of generalization. Most deep learning models are trained on physiological data of one anatomical region, which limits their applicability to pathological cases, which represent the scenarios most relevant to clinical use. Additionally, for complex tasks such as 3D PAT and USCT reconstruction, limited GPU memory may pose another constraint [41, 80]. Addressing this issue requires the development of more memory-efficient architectures for image reconstruction. Another well-known limitation in deep learning is the need for large training datasets, which may be particularly challenging for in vivo PAT or USCT data [137]. Some studies have supplemented missing data with synthetic datasets [29, 72, 77], but it is crucial that these accurately reflect real-world variations and artifacts to ensure effectiveness. Without robust training datasets, models risk poor generalizability and performance.

An important consideration for deep learning methods is the influence of hyperparameter settings, such as learning rate, batch size, and number of epochs, on model performance [138]. Due to the substantial heterogeneity across studies and inconsistent reporting practices, these aspects were not systematically analyzed in this review. Hyperparameter tuning remains highly method- and modality-dependent [138]. Given the intrinsic differences between imaging modalities, modality-specific hyperparameter optimization may be beneficial. Future research should aim to systematically evaluate and transparently report preprocessing protocols and hyperparameter configurations, particularly in relation to the imaging modality and task at hand.

### Iterative Reconstruction Algorithms

Iterative reconstruction refers to algorithmic techniques that iteratively refine image estimates by applying statistical and physical models to suppress noise while preserving spatial resolution and image quality [139]. Iterative reconstruction algorithms have been foundational in ultrasound-based tomographic imaging, but they face notable limitations compared to deep learning methods. These algorithms are computationally intensive, making them impractical for real-time imaging [140]. Consequently, although iterative methods remain valuable in specific contexts, recent literature increasingly favors deep learning approaches, which offer greater efficiency and flexibility.

### Synthetic Aperture Methods

Synthetic aperture techniques enhance resolution by combining signals from multiple transducer positions, effectively simulating a larger probe aperture [141]. All synthetic aperture methods were used for USCT imaging. A key challenge of synthetic aperture techniques is their sensitivity to sound-speed variations in tissue, which can distort images and blur boundaries between different tissue types, reducing image resolution [58, 60, 61, 64, 102].

### Fourier/Transform-Based Methods

Fourier-based methods reconstruct images by converting time-domain signals to the frequency domain, using analytical solutions like FBP [23, 55]. Fourier and transform-based methods, although sometimes used in ultrasound tomographic imaging, have inherent limitations. These methods depend on uniform data sampling and make simplified assumptions about wave propagation, which may restrict their ability to handle real-world complexities. Their performance further lags behind modern deep learning approaches, as they lack the flexibility to learn data-driven features [23, 55]. Consequently, Fourier and transform-based methods are less adaptable and effective in addressing the challenges of contemporary imaging needs and will likely see little development in the future.

### Future Perspectives

This review provides a detailed overview of methods for transforming ultrasound data into tomographic images, with a particular emphasis on the increasing reliance on deep learning approaches across various modalities. The field currently remains far from routine clinical application but definitely holds promise for clinical application. Among the most promising applications, breast imaging stands out due to the high prevalence of breast cancer and the need for non-invasive, radiation-free, high-quality imaging for routine screening and diagnostics. In neurological applications, ultrasound-based tomographic imaging could be especially valuable for younger patients or when applied intraoperatively, due to it not being constrained by ossified cranial sutures in these cases.

Musculoskeletal imaging is another area where this technology could prove to be advantageous. The ability to generate detailed 3D reconstructions of joints and bones could provide a cost-effective and efficient alternative to MRI in less complex cases, such as joint evaluations or minor fractures. Finally, the application of ultrasound-based tomographic imaging in low- and middle-income countries is particularly compelling. In these settings, where access to advanced imaging modalities such as CT and MRI is often limited, this technology could democratize access to high-quality diagnostics, addressing a critical gap in global healthcare equity.

While these applications hold great promise, ultrasound-based tomographic imaging remains in its early stages of development and faces several critical challenges. Key obstacles include the scarcity of robust and diverse datasets for training deep learning models, high computational requirements, difficulties in integrating these technologies with existing imaging systems, and a lack of proper validation and benchmarking [142, 143]. Before clinical integration can be considered, these models must first be optimized for efficiency and adaptability. Ideally, emerging deep learning architectures such as diffusion models and transformers, which have shown strong performance in related imaging tasks, should be leveraged. A targeted approach focusing on specific anatomical regions, such as the breast, brain, or musculoskeletal system, where the potential benefits would be particularly pronounced, should be prioritized. Once refined for these applications, the algorithms can then be expanded and generalized to perform reliably across diverse datasets and clinical conditions. Robust validation protocols are essential to ensure reliability and safety, including evaluations under both physiological and pathological conditions. Following this, large-scale, multicenter trials will be necessary to establish their clinical efficacy and safety.

Another important factor to consider is the choice of evaluation metrics. This review did not systematically collect data on these metrics, but heterogeneity was observed. Some studies relied on basic image similarity measures such as Dice scores, PSNR, or SSIM. Yet, no consistent use of metrics or framework for assessing reconstruction quality was available. Ideally, future research should determine which evaluation metrics are most appropriate for this task so that future methods can be evaluated on this basis and more effectively benchmarked against each other.

Furthermore, it is important to distinguish between algorithms designed for freehand ultrasound, which is prone to variability due to inconsistent probe movement, and those developed for fixed ultrasound systems, which benefit from controlled acquisition conditions. Ideally, future research should first focus on reconstruction algorithms for fixed ultrasound systems to lay the foundation for the more complex freehand ultrasound.

### Limitations

A notable limitation of this scoping review is the inadvertent bias introduced by the inclusion of search terms specifically related to deep learning techniques. This may have resulted in an overrepresentation of deep learning methods within the review. Furthermore, the search strategy employed during the reference screening process did not specifically target key imaging modalities, as terms such as “model-based,” “optimization-based,” and “full waveform inversion” were not included. This lack of comprehensive coverage may have led to the exclusion of critical papers that focus on these alternative methodologies, potentially limiting the scope and completeness of the review.

As this is a scoping review by design, it is also expected that some papers may have fallen outside the scope of our review. To mitigate gaps, reference screening was conducted, and preprint publications were included. Additionally, no systematic or quantitative assessment of the studies was conducted due to the significant heterogeneity across the included research.

## Conclusion

The synthesis of tomographic images from ultrasound represents a groundbreaking development in medical imaging, offering the potential to significantly enhance accessibility to high-quality diagnostic tools. This review highlights a clear shift from traditional approaches, such as iterative reconstruction methods, toward the adoption of deep learning models. However, these emerging methods face critical challenges, including a lack of reliable validation, high computational demands, and limited generalizability. Addressing these issues will require substantial efforts to improve the efficiency, accuracy, and robustness of these models before they can be considered viable for clinical implementation.

## Data Availability

The data in support of our findings can be obtained upon reasonable request from the corresponding author.

## Abbreviations

US: Ultrasound
CT: Computed tomography
LMIC: Low- and middle-income countries
MRI: Magnetic resonance imaging
GAN: Generative adversarial network
CNN: Convolutional neural network
2D: Two-dimensional
3D: Three-dimensional
PAT: Photoacoustic tomography
USCT: Ultrasound computed tomography
